# Shared and differential fractional amplitude of low-frequency fluctuation patterns at rest in major depressive disorders with or without sleep disturbance

**DOI:** 10.3389/fpsyg.2023.1153335

**Published:** 2023-03-22

**Authors:** Nanxi Zheng, Yangpan Ou, Huabing Li, Feng Liu, Guojun Xie, Ping Li, Bing Lang, Wenbin Guo

**Affiliations:** ^1^Department of Psychiatry, and National Clinical Research Center for Mental Disorders, The Second Xiangya Hospital of Central South University, Changsha, Hunan, China; ^2^Department of Radiology, The Second Xiangya Hospital of Central South University, Changsha, Hunan, China; ^3^Department of Radiology, Tianjin Medical University General Hospital, Tianjin, China; ^4^Department of Psychiatry, The Third People's Hospital of Foshan, Foshan, Guangdong, China; ^5^Department of Psychiatry, Qiqihar Medical University, Qiqihar, Heilongjiang, China

**Keywords:** fractional amplitude of low-frequency fluctuation, major depressive diorders, sleep disturbance, MRI, correlation

## Abstract

**Objective:**

Sleep disturbances (SD) are commonly found in patients with major depressive disorder (MDD). This study aims to explore the influence of SD symptoms on clinical characteristics in patients with MDD and to investigate the shared and distinct fractional amplitude of low-frequency fluctuation (fALFF) patterns in these patients with or without SD symptoms.

**Methods:**

Twenty-four MDD patients with SD symptoms (*Pa_s*), 33 MDD patients without SD symptoms (*Pa_ns*) and 32 healthy controls (HCs) were included in this study. The fALFF and correlation analyses were applied to analyze the features of imaging and clinical data.

**Results:**

*Pa_s* showed more severe anxiety and depression than *Pa_ns*. Compared with *Pa_ns*, *Pa_s* exhibited increased fALFF value in the left precuneus. Patients shared abnormal fALFF in the frontal-occipital brain regions. There was a positive correlation between fALFF values of the left precuneus and sleep disturbance scores (*r* = 0.607, *p* = 0.0000056734) in all patients in addition to a negative correlation between fALFF values of the left MOG/cuneus and HAMD-17 total scores (*r* = −0.595, *p* = 0.002141) in *Pa_s*. The receiver operating characteristic (ROC) results of the fALFF could be used to discriminate *Pa_s* from *Pa_ns* with a specificity of 72.73% and a sensitivity of 70.83%.

**Conclusion:**

*Pa_s* displayed more serious anxiety and depression symptoms. Patients shared abnormal fALFF in the frontal-occipital brain regions, which may be a common characteristic for MDD. And increased fALFF value in the left precuneus might be a specific neuroimaging feature of MDD patients with SD symptoms.

## Introduction

Major depressive disorder (MDD) is a worldwide disabling disease which brings remarkable social and economic burden. Approximately over 300 million people around the world suffer depression and the prevalence is still rising (Friedrich, 2017). MDD is characterized by persistently depressive mood, anxiety, cognitive impairment, sleep disorders, suicidal thoughts and motivation alteration. About 60% patients with MDD will have recurrences with 10–20% risk of ineffective remission using current therapies in continuous episodes (Monroe & Harkness, 2011). Due to its early onset and frequent recurrences, MDD is one of the most important causes of social disability (CADTH, 2016).

Sleep disturbances (SD) are commonly reported in patients with persistent depression (Clark et al., 2009). Over 90% patients with MDD take SD as the chief complaint. The usual performance of SD includes difficulty of falling asleep, sleep interruption and early wake-up (Tsuno et al., 2005). SD could impair emotion, memory, attention and other executive functions (Liu et al., 2015). But SD often precedes depression rather than secondary to it, and constantly exists in the remission period. SD also affects the development, treatment response and prognosis of depressive disorder, and increases the risk of depression development (Lovato & Gradisar, 2014). Improvement of sleeping in patients with depression could remiss the depressive symptoms (Manber et al., 2008). A meta-analysis has suggested that people with insomnia have doubled risk of suffering depression than those without SD (Baglioni et al., 2011).

Apparently, there is a potentially close correlation between MDD and SD, which is not simply a cause-effect relationship but appears to be a complicated bidirectional association. Recently, neuroimaging has become a valuable way to investigate the pathogenesis and mechanisms of mental disorders. Amplitude of low-frequency fluctuation (ALFF) and fractional ALFF (fALFF), which are based on the blood oxygenation level-dependent (BOLD) fMRI signals, have been widely applied to identify the low-frequency fluctuation of spontaneous neural activity at rest (Biswal et al., 1995; Zhuo et al., 2019). In recent years, multiple studies have explored the underlying association between SD and MDD. A recent research has found that there was a correlation between the severity of insomnia in patients with MDD and increased ALFF values in the right inferior frontal gyrus (IFG)/anterior insula (Liu et al., 2018). And a positive correlation between fALFF values of the right superior parietal gyrus (SPG) and baseline sleep efficiency in patients with MDD was also reported (Chen et al., 2022). Another study has indicated that the SD scores of the 17-item Hamilton Depression Rating Scale for Depression (HAMD-17) could be predicted by a combination of gray matter density and fALFF values (Shi et al., 2021). And the smaller cortex surface area was found in frontoparietal cortices including the left inferior frontal gyrus pars triangularis, left frontal pole, right superior parietal cortex, and right supramarginal gyrus in patients with MDD with serious insomnia (Leerssen et al., 2020). Besides, functional connectivity between nucleus accumbens and default-mode network (DMN) was associated with the severity of insomnia, and nucleus accumbens-based functional connectivity in the reward network was correlated with depressive symptoms in patients with chronic insomnia (Gong et al., 2021). However, it remains unclear whether there were shared and differential brain spontaneous neural activities at rest in MDD with or without SD.

In the present study, we aimed to determine the clinical characteristics of MDD patients with or without SD symptoms. Furthermore, we have employed fALFF to analyze the common and different alterations of brain spontaneous neural activities in these two groups in order to provide more insights to better understand correlation between MDD and SD.

## Methods

### Participants

The patients with MDD were recruited from the Second Xiangya Hospital, and the HCs were recruited from the local community. All participants were age-and education-matched and were Han Chinese and right-handed. The HCs would be ruled out if they: (1) had acute physical illness (here only included structural or organic diseases) or neurological illness, or a history of substance abuse; (2) had a history of brain injury resulting in loss of consciousness; (3) were pregnant or were unable to undergo MRI scans.” The diagnosis of MDD was based on the Diagnostic and Statistical Manual of Mental Disorders-Fifth Edition (DSM-5) by two psychiatrists independently. Patients with MDD were allocated to *Pa_s* group (patients with chief complaint of SD symptoms, and SD scores >4, *n* = 26) and *Pa_ns* group (patients without chief complaint of SD symptoms, and SD scores ≤4, *n* = 34) depending on the SD scores which were computed by adding scores of items 4, 5, and 6 of the 17-item Hamilton Rating Scale for Depression (HAMD-17) (Liu et al., 2018). Detailed demographic information was shown in Table 1. All patients had HAMD-17 scores >20 and had no history of major somatic diseases or other psychiatric disorders, no history of antidepressant treatment, substance abuse or electroconvulsive therapy. Pregnancy or incapacity of participating in brain MRI scan was also excluded.

**Table 1:** Demographic and clinical characteristics of participants.

Variables	*Pa_s* group (*n* = 24)	*Pa_ns* group (*n* = 33)	HCs (*n* = 32)	*F*/χ^2^/*t*	*Post hoc t*-tests or *p*/*t* values
Age (years)	31.375 ± 6.78	29.48 ± 7.13	29.59 ± 5.00	1.07^a^	0.35
Sex (male/female)	12/12	6/27	15/17	8.09^b^	0.02
Education (years)	13.63 ± 3.73	13.91 ± 3.06	14.59 ± 2.82	0.72^a^	0.49
Illness duration (months)	5.83 ± 4.12	6.77 ± 4.65	–	0.78^c^	0.43
BAI scores	47.39 ± 13.11	37.97 ± 7.58	22.63 ± 2.28	63.75^a^	*Pa_s* > *Pa_ns* > HCs
HAMD-17 scores	23.38 ± 3.70	20.18 ± 2.64	0.94 ± 0.95	670.29^a^	*Pa_s* > *Pa_ns* > HCs
Sleep disturbances^*^	5.54 ± 0.51	3.15 ± 0.94	0.34 ± 0.60	357.41^a^	*Pa_s* > *Pa_ns* > HCs
Anxiety/Somatization	7.38 ± 1.91	6.76 ± 1.82	0.44 ± 0.62	190.43^a^	*Pa_s*, *Pa_ns* > HCs
Retardation symptoms	6.25 ± 1.51	6.64 ± 1.32	0.16 ± 0.37	313.83^a^	*Pa_s*, *Pa_ns* > HCs
Weight loss	0.71 ± 0.81	0.39 ± 0.70	0	9.83^a^	*Pa_s*, *Pa_ns* > HCs
Cognitive disturbances	3.50 ± 2.04	3.24 ± 1.70	0	52.83^a^	*Pa_s*, *Pa_ns* > HCs

Data was displayed with mean ± standard deviation. HAMD-17_,_ the 17-Item Hamilton Rating Scale for Depression; BAI, Beck anxiety inventory; Pa_s, major depressive disorder with sleep disturbance; Pa_ns, major depressive disorder without sleep disturbance; HCs, healthy controls.

a ANOVA.

b Chi-square test.

c Two sample *t*-test.

* Sleep disturbance scores were computed by adding scores of items 4, 5, and 6 of the HAMD-17 scale.

The study was conducted according to the Helsinki Declaration and approved by the Medical Research Ethics Committee of the Second Xiangya Hospital, Central South University, Changsha, China. All participants signed a written informed consent.

### Assessment tools

The severity of depressive symptoms was assessed by the scores of HAMD-17. Anxiety/somatization severity was evaluated by items10-13, 15 and 17. Retardation symptoms were evaluated by items 1, 7, 8 and 14. Severity of cognitive disturbances was evaluated by items 2, 3 and 9. Severity of weight loss was evaluated by items 16. Anxiety state was assessed by the Beck anxiety inventory (BAI).

### Image acquisition

All participants received the rs-fMRI scanning on a 3.0 T scanner (General Electric, FairfieldConnecticut, USA). They were informed to lay supine in the scanner with heads fixed with a foam padding and belt, keeping motionless with eyes closed. Echo planar imaging (EPI) was employed to acquire the resting-state functional images with the following parameters: repetition time/echo time (TR/TE) = 2000/30 ms 33 axial slices, 64 × 64 matrix, 90° flip angle, 22 cm FOV, 4 mm section thickness, no slice gap, and 240 volumes.

### Imaging data processing

Data were pre-processed using the Data Processing Assistant for Resting-State fMRI (DPARSF v5.2; DPARSF^1^) software (Chao-Gan & Yu-Feng, 2010). The first 10 images were deleted for MRI to achieve signal equilibrium and for the participants to adapt to the scanning noise. And the resting images were corrected of slice timing and head motion. We excluded participants with head motion exceeding 2 mm of displacement in the x-, y-, or z-axis or 2^°^ angular motion in each axis. These images were normalized to the standard Montreal Neurological Institute (MNI) space and resampled with a resolution of 3 × 3 × 3 mm^3^. Spatial smoothing was conducted *via* a 4-mm Gaussian kernel of full width at half maximum (FWHM). Linear trend subtraction and temporal filtering (0.01–0.08 Hz) were performed on the time series of each voxel to reduce the effect of low-frequency drifts and physiological high frequency respiratory and cardiac noise for further analysis. Calculation of fALFF was refered to the previous study (Zou et al., 2008). Fast Fourier transform was applied to convert the time course of each voxel to the frequency domain to obtain the power spectrum. Then, the square root of the power spectrum was calculated, and the average was obtained across 0.01–0.08 Hz. The fALFF was calculated as the ratio of the sum of amplitude across 0.01–0.08 Hz to that across the complete frequency range. For standardization, the fALFF in each voxel was divided by the global mean fALFF value.

### Statistical analysis

Difference in demographic, clinical and neuroimaging data across *Pa_s*, *Pa_ns* and HCs was compared. The continuous data were compared by Student’s *t*-test or one-way analysis of variance (ANOVA) and the categorical data were compared with chi-square test.

Analyses of covariance (ANCOVA), followed by *post-hoc t*-tests, was performed on fALFF maps of each participant across the three groups to discriminate the group differences. Age, sex, years of education and framewise displacement were applied as covariates. The results were FDR (false discovery rate) corrected at *p* < 0.05.

Correlation analysis was performed in the fALFF values of clusters with significant difference. Pearson or Spearman correlation analyses were used to assess the correlation between the extracted fALFF and scores of HAMD-17 and BAI scales, follwed by the Bonferroni correction to raise the inspection level.

The receiver operating characteristic (ROC) was used to discriminate Pa_s from Pa_ns. And the best cutoff which maxmized the sum of sensitivity and specificity was calculated.

## Results

### Demographic and clinical characteristics

We have recruited sixty first-episode patients with MDD at the Second Xiangya hospital, and 34 HCs from the local community. We, respectively, excluded 2, 1 and 2 participants from *Pa_s*, *Pa_ns* and HCs due to the head motion. Finally, 24 *Pa_s*, 33 *Pa_ns* and 32 HCs were included in the final analysis. The detailed data were shown in Table 1. No difference was found in age and years of education among the three groups except gender. And there was no difference of illness duration between *Pa_s* and *Pa_ns*. Meanwhile, *Pa_s* had higher scores in BAI scale, HAMD-17 scale and sleep disturbance than *Pa_ns*. All patients had higher scores in BAI scale, HAMD-17 scale and five other subscales than HCs.

### Difference in fALFF across groups

**Figure 1:**
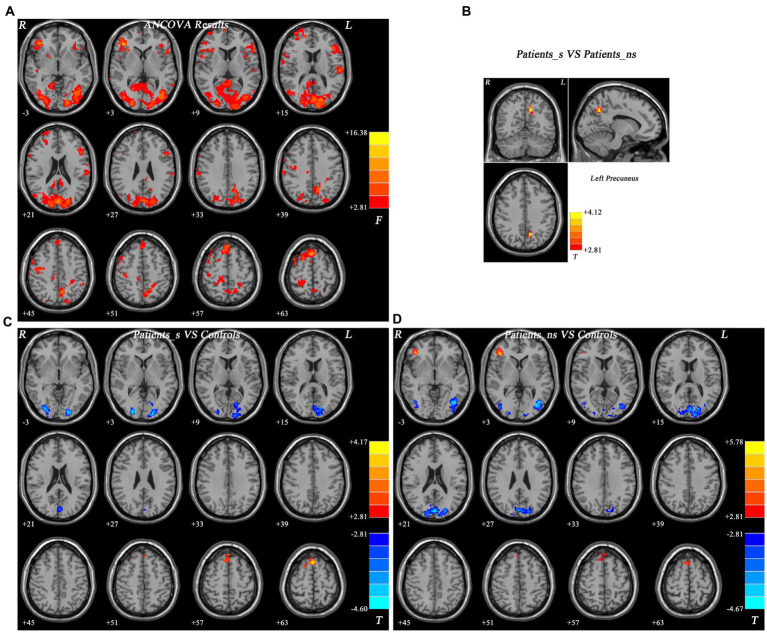
Different fALF*F* values and brain regions with significantly different fALFF values across three groups. The color bar indicates the *F* values based on ANCOVA **(A)**. Brain regions with fALFF difference between *Pa_s* and *Pa_ns* **(B)**. Brain regions with fALFF difference between *Pa_s* and HCs **(C)**. Brain regions with fALFF difference between *Pa_ns* and HCs **(D)**. For panels **(B–D)**, the color bar indicates the *t* values from *post-hoc t*-tests. Red and blue colors, respectively, represent increased and decreased fALFF. fALFF, fractional amplitude of low-frequency fluctuation; ANCOVA, analysis of covariance. *Pa_s*, major depressive disorder with sleep disturbance; *Pa_ns*, major depressive disorder without sleep disturbance. HCs, healthy controls.

The fALFF values were collected and compared with ANCOVA analyses and significant differences were found in the frontal, occipital, and parietal gyri among the three groups (Figure 1A).

Compared with *Pa_ns*, *Pa_s* showed increased fALFF values in the left precuneus (Figure 1B, Table 2). Besides, *Pa_s* exhibited higher fALFF values in the bilateral superior MPFC/SMA but lower values in the right middle occipital gyrus (MOG) /fusiform gyrus and left MOG/cuneus relative to HCs (Figure 1C, Table 2). In addition, increased fALFF values were found in the right inferior frontal gyrus (IFG) and bilateral superior MPFC/SMA in *Pa_ns* compared to HCs. And *Pa_ns* showed decreased fALFF values in the right MOG/inferior occipital gyrus (IOG), left MOG/IOG and bilateral MOG/cuneus than HCs (Figure 1D, Table 2).

**Table 2:** Significant fALFF differences across three groups.

Cluster location	Peak (MNI)			Number of voxels	*T* value
	*x*	*y*	*z*		
Pa_s vs Pa_ns					
Left Precuneus	−12	−60	39	55	4.1244
Pa_s vs HCs					
Bilateral Superior MPFC/SMA	0	21	63	135	4.1694
Right Middle Occipital Gyrus/Fusiform Gyrus	24	−93	0	172	−4.5984
Left Middle Occipital Gyrus/Cuneus	−21	−93	−3	278	−4.4966
Pa_ns vs HCs					
Right Inferior Frontal Gyrus	42	36	0	103	5.7803
Bilateral Superior MPFC/SMA	9	15	66	55	4.0617
Right Middle Occipital Gyrus/Inferior Occipital Gyrus	42	−69	−3	93	−3.6609
Left Middle Occipital Gyrus/Inferior Occipital Gyrus	−42	−69	0	269	−4.5238
Bilateral Middle Occipital Gyrus/Cuneus	0	−87	21	438	−4.4909

MNI, Montreal Neurological Institute; fALFF, fractional amplitude of low-frequency fluctuation; MPFC, medial prefrontal cortex; SMA, supplementary motor area. *Pa_s*, major depressive disorder with sleep disturbance; *Pa_ns*, major depressive disorder without sleep disturbance; HCs, healthy controls.

### Correlations

**Figure 2:**
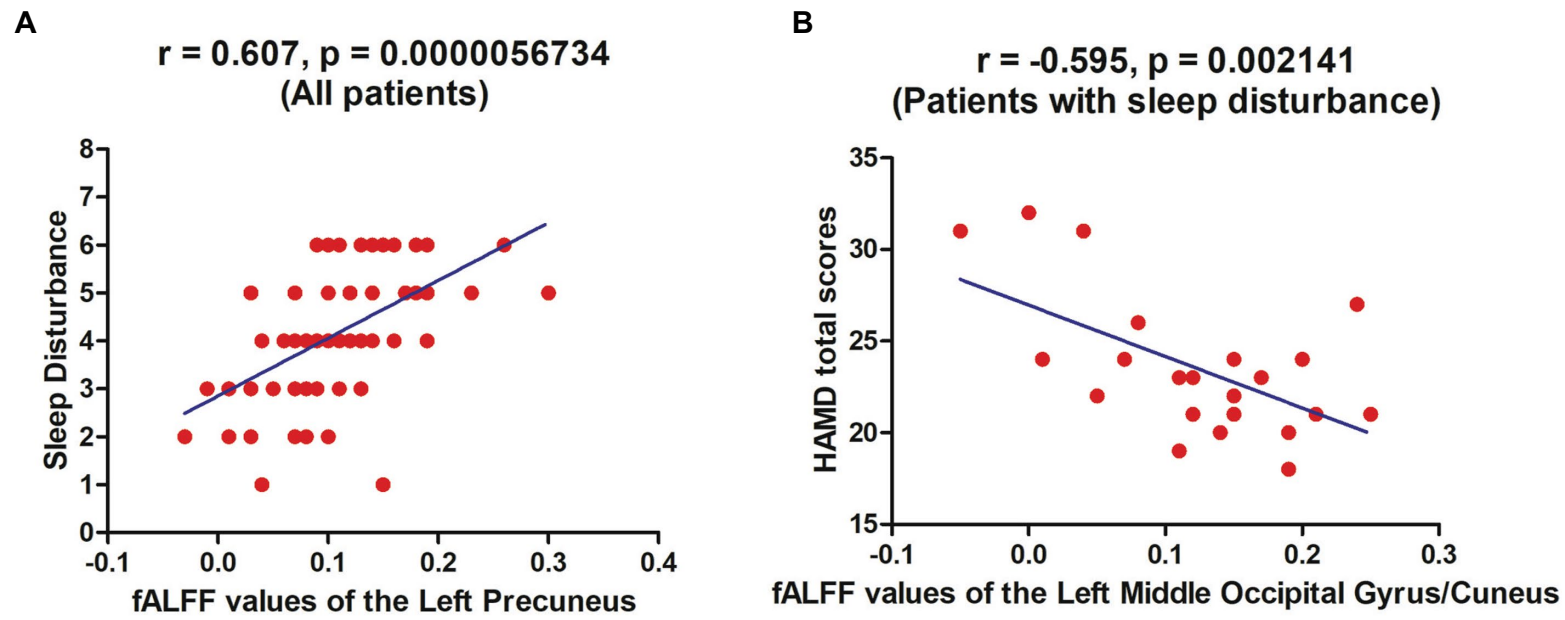
Correlations between fALFF values and clinical variables. For all patients with MDD, there was a positive correlation between fALFF values of the left precuneus and BAI scores **(A)**. For *Pa_s*, there was a negative correlation between fALFF values of the left middle occipital gyrus/cuneus and HAMD-17 total scores **(B)**. fALFF, fractional amplitude of low-frequency fluctuation; MDD, major depressive disorder; BAI, Beck anxiety inventory; *Pa_s*, major depressive disorder with sleep disturbance; HAMD-17, 17-item Hamilton Rating Scale for Depression.

For all patients, there was a positive correlation between fALFF values of the left precuneus and BAI scores (*r* = 0.308, *p* = 0.023) in addition to fALFF values of the left precuneus and sleep disturbance scores (*r* = 0.607, *p* = 0.0000056734) (Figure 2A). But the correlation between fALFF values of the left precuneus and BAI scores failed to survive the Bonferroni correction.

For *Pa_s*, the fALFF values of the right MOG/fusion gyrus and HAMD-17 total scores (*r* = −0.406, *p* = 0.049), fALFF values of the left MOG/cuneus and HAMD-17 total scores (*r* = −0.595, *p* = 0.002141) (Figure 2B) apart from fALFF values of the left MOG/cuneus and cognitive disturbance scores (*r* = −0.515, *p* = 0.010) displayed inverse correlations. But only the correlation between fALFF values of the left MOG/cuneus and HAMD-17 total scores survived the Bonferroni correction.

For *Pa_ns*, positive correlations were found between weight lost scores and fALFF values of the bilateral MOG/cuneus (*r* = 0.379, *p* = 0.030) or the left MOG/IOG (*r* = 0.492, *p* = 0.004) or the right MOG/IOG (*r* = 0.426, *p* = 0.013). But all these correlations did not pertain after the Bonferroni correction.

### ROC results

**Figure 3:**
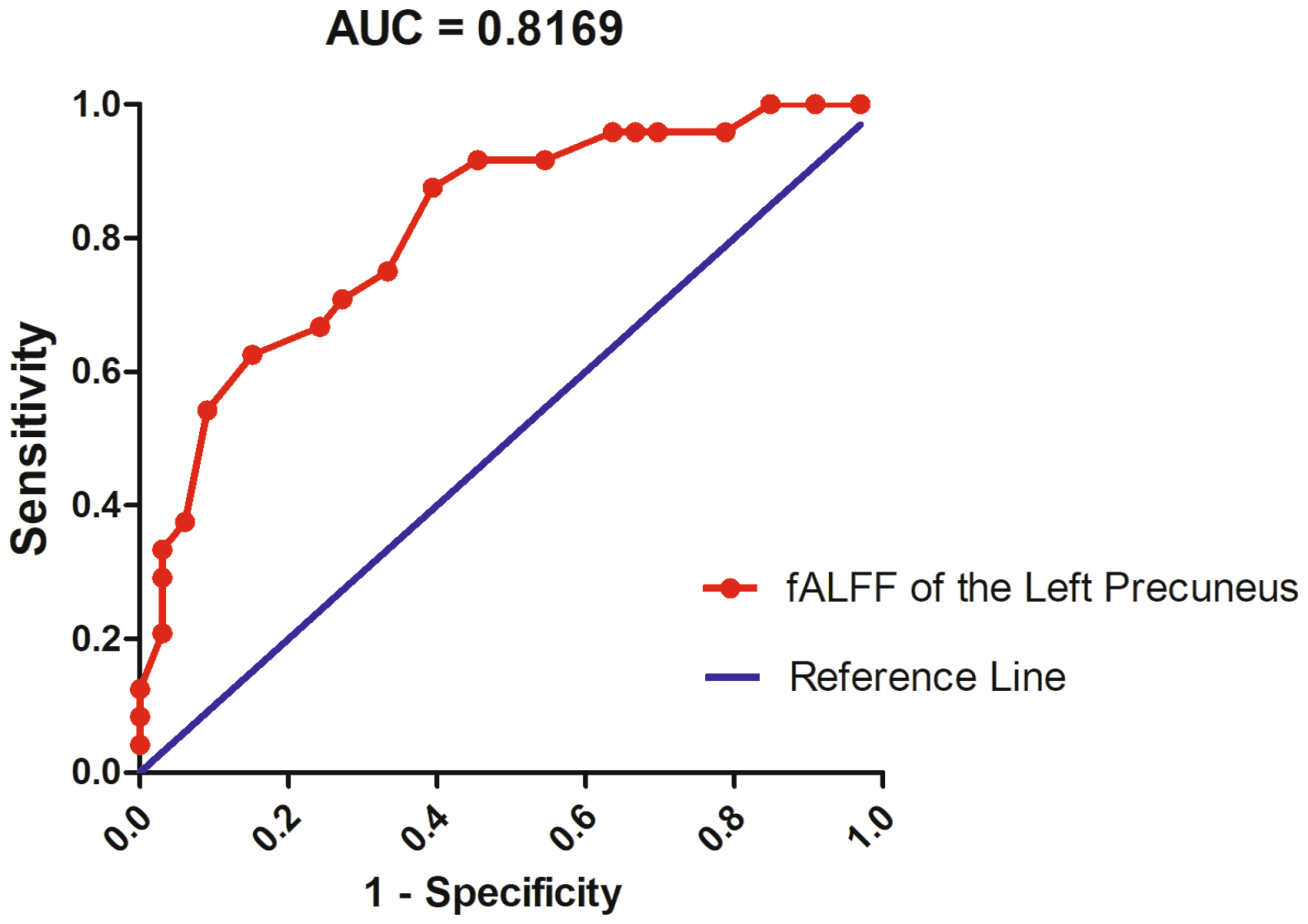
Receiver operating characteristic (ROC) results indicated that fALFF values in the left precuneus could discriminate *Pa_s* from *Pa_ns*. *Pa_s*, major depressive disorder with sleep disturbance; *Pa_ns,* major depressive disorder without sleep disturbance.

The fALFF values in the left precuneus of *Pa_s* and *Pa_ns* were further analysed with the receiver operating characteristic (ROC). The results indicated that fALFF values in the left precuneus could be used to differentiate *Pa_n* from *Pa_ns* with a satisfactory specificity of 72.73% and a sensitivity of 70.83% (Figure 3). The area under the curve (AUC) was 0.8169 for the ROC results.

## Discussion

In this study, we found that *Pa_s* had more severe anxiety and depression than *Pa_ns*, which could be reflected with the higher scores of BAI scale and HAMD-17 scale *Pa_s* obtained. This suggested that SD symptoms negatively influenced MDD patients. In addition, patients shared abnormal fALFF in the frontal-occipital brain regions, which may be a common characteristic for MDD. Moreover, *Pa_s* showed increased fALFF values in the left precuneus than *Pa_ns*, which could be used to discriminate the two groups according to the ROC results with a specificity of 72.73% and a sensitivity of 70.83% (Figure 3).

This study also showed that *Pa_s* presented higher scores of BAI scale and HAMD-17 scale relative to *Pa_ns*. But there was no significant difference in the scores of anxiety/somatization, retardation symptoms, weight loss and cognitive disturbances between these two groups. Despite *Pa_s* did not have a higher score of anxiety/somatization in HAMD-17, they did score higher in BAI scale, which may be due to the discrepant abilities to detect statistical differences in anxiety. BAI scale has a total of 21 items to assess anxiety severity. It focuses on somatic symptoms of anxiety and is applied to acquire purer measure of anxiety which discriminates from depression (Julian, 2011). While anxiety/somatization factor in HAMD-17 is unstable and is weakly correlated to Hamilton Anxiety Scale (HAMA), which may not be enough to measure anxiety severity in depressive patients (Goldberger et al., 2011). This means BAI scores of anxiety could be more reliable relative to anxiety/somatization scores in HAMD-17. These results suggested that *Pa_s* had a more severe total disease state, which indicated that SD was related to higher levels of anxiety and depression. As we know, SD is a common symptom in MDD which is used to help MDD diagnosis in DSM-5(Spiegel et al., 2013). SD is also considered to be a main risk factor and predictor of depression (Baglioni et al., 2011). Recently, mounting evidence has demonstrated that SD occurs prior to depression (Jaussent et al., 2011). An early cross-sectional study suggested that young adults who had persistent insomnia presented more severe major depression and generalized anxiety (Vollrath et al., 1989). In addition, depressive patients with SD usually had more difficulties in treatment and continuous insomnia was thought to be an important predictor of depression relapse (Vollrath et al., 1989). All these implied that there may be a bidirectional relationship between SD and MDD. Increasing evidence has shown that elevated levels of inflammatory cytokines like IL-6 and TNFα were found in adults with MDD and people with sleep disorders (Irwin et al., 2006; Dowlati et al., 2010). SD could result in increased inflammation whereas antagonism of endogenous inflammation would improve depressive symptoms (Irwin et al., 2006,  2010; Raison et al., 2013). But the exact interaction between them is still unknown. Besides, twin studies suggest that MDD and SD are all heritable and other research demonstrated that they overlap significantly (Lind et al., 2015, 2017; Fang et al., 2019). Due to the close association between SD and MDD, improved SD would be beneficial to the treatment and prognosis of MDD.

*Pa_s* showed higher fALFF values in the left precuneus compared with *Pa_ns*. And for all patients with MDD, there was a positive correlation between fALFF values in the left precuneus and SD scores. These results indicate that the left precuneus might be closely associated with SD symptoms in MDD. The precuneus plays an important role in highly integrated cognitive tasks including attention, conscious perception, visuospatial imagery, episodic and working memory retrieval (Lundstrom et al., 2005; Cavanna & Trimble, 2006). And precuneus is a key component of the default-mode network (DMN) which mainly spans medial prefrontal cortex, medial temporal cortex and posterior cingulate cortex (Fox & Raichle, 2007; Buckner et al., 2008). It is reported that the DMN is related to information collection, self-referential mental activity, consciousness, adaption, mind wandering or daydreaming, emotion and anxiety (Gusnard et al., 2001a, b; Simpson et al., 2001; Cabeza et al., 2002; Mason et al., 2007; Andrews-Hanna et al., 2010; Wilson et al., 2010). When brain is resting, DMN is activated to maintain internal mental state. Plentiful studies have suggested that patients with sleep disorders have functional alteration in the DMN. For example, Luo et al. have found increased functional connectivity in the left precuneus of patients with sleep disorders and mild cognitive impairment (Luo et al., 2022). In addition, decreased functional connectvity is concerned with dysfunctional cognition in the DMN at rest and enhanced functional connectivity may be compensatory for the impaired cognition (Luo et al., 2022). There was no significant difference in cognitive disturbance between *Pa_s* and *Pa_ns*. Therefore, the abnormally increased fALFF values in the left precuneus might reflect SD-associated impairment in cognition along with other functions and an adaptive compensation in the DMN (Chen et al., 2016).

Compared to HCs, the *Pa_s* and *Pa_ns* patients presented increased fALFF values of the bilateral superior MPFC/SMA. MPFC is of great importance in attention, working memory, long-term memory and emotional and inhibitory control (Bittar & Labonté, 2021) and when dysfunctional, may lead to the depressive-like behaviors. MPFC has been considered to be closely related with MDD (Belleau et al., 2019). In other studies, MPFC of patients with MDD showed increased ALFF values (Gong et al., 2020) and the altered values were positively associated with glutamate concentration in the MPFC (Zhang et al., 2016). The raised levels of glutamate in prefrontal cortex of patients with MDD were confirmed in post-mortem studies (Hashimoto et al., 2007). In addition, numerous evidence has suggested that dysregulated glutamate-glutamine cycling in the MPFC is related to MDD. Inhibition of glutamine synthase which could convert glutamate to glutamine in MPFC would cause depressive behavior (Hashimoto et al., 2007; Lee et al., 2013). Apart from changes in glutamate concentration, decreased GABA levels in MPFC were found in patients with MDD which could be elevated by effective treatment (Dubin et al., 2016; Brennan et al., 2017). Furthermore, the reduced GABA levels in MPFC are correlated with treatment resistance in MDD (Price et al., 2009; Levinson et al., 2010). Several meta-analyses have reported that patients with MDD displayed reduced MPFC volume. And the reduction of volumes is more significant when depression could not remit (Frodl et al., 2008). Thus, these findings may suggest that the abnormally increased fALFF value in the MPFC is a potential marker for MDD.

Compared with HCs, *Pa_s* showed lower fALFF values in the right MOG/fusiform gyrus and the left MOG/cuneus. *Pa_ns* also displayed lower fALFF values in the right MOG/IOG, left MOG/IOG and bilateral MOG/cuneus. Besides, fALFF values of the left MOG/cuneus in *Pa_s* were negatively correlated to total HAMD-17 scores. Various research has indicated that the occipital cortex may be associated with MDD (Bhagwagar et al., 2007; Furey et al., 2013). Zhao et al. have found thicker gray matter in the left fusiform and right lateral occipital cortex and thinner gray matter in the bilateral lingual cortex and left cuneus of patients with MDD (Zhao et al., 2017). Lee et al. and Na et al. found thinner left occipital cortex and bilateral fusiform gyrus in patients with MDD (Na et al., 2016; Lee et al., 2021). Moreover, occipital bending that occipital cortex wraps around other brain areas might be a characteristic of MDD which often exists in treatment resistant patients with MDD (Siebert, 2015). Patients with MDD showed frequent structural abnormalities in regions of visual recogniton network heavily involved in facial emotional processing. And these structural alteration within the visual recognition network might be related to damaged selective attention in MDD (Desseilles et al., 2009; Tao et al., 2013; Zhao et al., 2017). Negative attention bias in the information processing is of great importance in depression episode which could contribute directly to depression and serve as a risk factor (Koster et al., 2009; Disner et al., 2011; Foland-Ross & Gotlib, 2012). Our previous research and Teng et al. respectively found decreased ALFF values of the occipital cortex and left-MOG in patients with MDD, indicating that visual processing was disturbed in MDD (Guo et al., 2012; Teng et al., 2018). Together with these studies, the low activity in the occipital cortex was possibly associated with dysfunctional visual emotional information processing in patients with MDD.

*Pa_ns* showed increased fALFF values in the right IFG relative to HCs. IFG is associated with emotion regulation and cognition control (Jastorff et al., 2016; Urgesi et al., 2016). Abnormal recruitment of IFG may be involved in emotional stimulus processing in adults with MDD (Disner et al., 2011). Our study included more females in *Pa_ns*. The increased fALFF values in the right IFG may be a result caused by the gender bias. It’s reported that MDD female patients showed greater hyperactivity in the right IFG than the left part during facial emotion processing. And this laterality was associated with patients’ performance (Briceño et al., 2013). There were many studies indicating that structurally altered IFG was related to MDD. For example, IFG volumes of patients with MDD were smaller than healthy people and had significant relation to depressive severity (Kandilarova et al., 2019; Dai et al., 2020). Thus, IFG may have some specific association with MDD about emotion processing, which shall be warranted in future studies.

There are still a few limitations which should be noticed in this study. First, our sample size was small and we did not classify different SD symptoms in detail to explore the fALFF or score distinction among patients with distinct SD performance. Second, this was a cross-sectional study which could not analyze cause-effect relationship thus we cannot define whether the abnormal fALFF values were the consequence or causation of SD in MDD. Third, our findings were limited in Han Chinese, and further studies in different ethnic groups are essential to validate this research.

## Conclusion

Our study has revealed that SD symptoms may produce negative effects in MDD patients. In addition, the shared and different fALFF changes in patients with MDD with or without SD symptoms were also demonstrated. Patients shared abnormal fALFF in the frontal-occipital brain regions, which may be a common characteristic for MDD. *Pa_s* showed increased fALFF value in the left precuneus compared with *Pa_ns*, which could be used to discriminate MDD patients with or without SD symptoms, indicating a potential abnormal activity within this region in MDD patients with SD symptoms.

## Data availability statement

The original contributions presented in the study are included in the article/supplementary material, further inquiries can be directed to the corresponding authors.

## Ethics statement

The studies involving human participants were reviewed and approved by Medical Research Ethics Committee of the Second Xiangya Hospital. The patients/participants provided their written informed consent to participate in this study.

## Author contributions

WG and BL designed the research. YO carried out the experiments and analyzed the data. WG, BL, and NZ wrote the paper. HL, FL, GX, and PL contributed to the MRI data acquisition. All authors contributed to the article and approved the submitted version.

## Funding

This study was supported by grants from the National Natural Science Foundation of China (Grant Numbers: 82171508 and 82071507).

## Conflict of interest

The authors declare that the research was conducted in the absence of any commercial or financial relationships that could be construed as a potential conflict of interest.

## Publisher’s note

All claims expressed in this article are solely those of the authors and do not necessarily represent those of their affiliated organizations, or those of the publisher, the editors and the reviewers. Any product that may be evaluated in this article, or claim that may be made by its manufacturer, is not guaranteed or endorsed by the publisher.

## Footnotes

^1^http://rfmri.org/
